# Supplementary material for: The Effect of Zinc, Selenium, and Their Combined Supplementation on Androgen Receptor Protein Expression in the Prostate Lobes and Serum Steroid Hormone Concentrations of Wistar Rats
Source: Nutrients. 2020 Jan 6;12(1):153. doi: 10.3390/nu12010153 (PMC7019230; doi:10.3390/nu12010153)
Supplement: Supplementary file 1 [file nutrients-12-00153-s001.pdf]

Supplementary Table S1. Blood Zn and Se concentrations (mean ± SD) after 30 and 90-day administration of Zn and/or Se, and after 90 days exposure with an additional 90-day post-administration period.

		Zn [μg/mL]	Se [μg/mL]
**30 days of administration**	**Control**	5.59±0.16	0.50±0.04
	**Zn**	5.87±0.24	0.51±0.03
	**Se**	5.69±0.24	0.55±0.03
	**Zn+Se**	5.72±0.26	0.52±0.02
**90 days of administration**	**Control**	5.41±0.21	0.49±0.02
	**Zn**	5.81±0.21	0.50±0.02
	**Se**	5.72±0.28	**0.57±0.03***
	**Zn+Se**	5.76±0.29	0.53±0.03
**90 days of administration and 90-day post administration period**	**Control**	5.19±0.33	0.50±0.02
	**Zn**	5.79±0.32	0.51±0.02
	**Se**	5.61±0.32	0.56±0.03
	**Zn+Se**	5.80±0.39	0.54±0.03

* result statistically significant in comparison to control group, $p \leq 0.05$
